# Fernbeschulung während der COVID-19 bedingten Schulschließungen weiterführender Schulen: Analysen zum Gelingen aus Sicht von Schülerinnen und Schülern

**DOI:** 10.1007/s11618-021-01006-7

**Published:** 2021-03-26

**Authors:** Ivo Züchner, Hannah Rahel Jäkel

**Affiliations:** FB 21, Institut für Erziehungswissenschaft, Philipps-Universität Marburg, Wilhelm-Röpke-Straße 6B, 35032 Marburg, Deutschland

**Keywords:** Fernbeschulung, Lernen, Digitale Medien, Distance education, E‑learning, COVID-19, Homeschooling, COVID-19, Digital media, Distance education, E‑learning, Homeschooling, Learning

## Abstract

Im Zuge der Schulschließungen im Rahmen der Schutzmaßnahmen gegen COVID-19 standen deutsche Schulen historisch erstmals vor der Aufgabe, flächendeckende Fernbeschulung für Schülerinnen und Schüler zu gewährleisten. Der über Nacht einsetzenden Herausforderung zur Konzeptionierung und Durchführung dieses Unterrichts auf Distanz begegneten Schulen, Lehrkräfte und Lernende in vielfacher Weise, ohne auf bewährte Strukturen zurückgreifen zu können. Der Beitrag entwirft anhand des Angebots-Nutzungs-Modells von Helmke (2012) und ersten Forschungsergebnissen zur Lehre während der Schulschließungen ein theoretisches Modell des Bedingungsgefüges von Fernbeschulung. Dieses wird genutzt, um dann anhand der Daten einer Online-Befragung mit 1128 Schülerinnen und Schülern von weiterführenden Schulen vom Mai 2020 exemplarisch anhand der von den Schülerinnen und Schülern eingeschätzten Fähigkeit zur Bewältigung der „Haus“Aufgaben das Zusammenspiel der skizzierten Dimensionen während der Fernbeschulung – mit besonderem Fokus auf die Rolle digitaler Medien.

## Abstract (en)

In the course of the school closings as part of the protective measures against COVID-19, German schools historically for the first time had the task of ensuring comprehensive distance education for all their pupils. Schools, teachers and students met this challenge of distance education with different practices, without being able to draw on former experience. In the paper, a theoretical framework for analyzing school distance education is developed based on Helmke’s “Angebots-Nutzungs-Modell” and research from the new situation. It serves as a base for an empirical analysis of the interrelationship between factors influencing the accomplishment of homework with data from a study with 1128 students from May 2020, with lays special emphasis on the use of digital media.

Im Zuge der Maßnahmen zur Eindämmung von COVID-19 wurden in Deutschland im März 2020 innerhalb kürzester Zeit alle Schulen bis auf Weiteres geschlossen (vgl. Anger et al. 2020a). So standen die Schulen von heute auf morgen vor der Herausforderung, einen Lehrbetrieb auf Distanz zu gewährleisten – ein Zustand, der für alle bis Mitte April 2020, für die meisten Klassenstufen darüber hinaus bis in den Mai anhielt und zum Jahresanfang 2021 nach Lockerungen im Herbst wieder einsetzte. Diese flächendeckende Beschulung zu Hause hat das deutsche Schulsystem im März 2020 völlig unerwartet getroffen und von Land zu Land, von Schule zu Schule und auch von Lehrkraft zu Lehrkraft wurden zur Fernbeschulung verschiedene Lösungen und Arbeitsansätze gefunden. Der Beitrag untersucht diese Fernbeschulung aus Sicht der Kinder und Jugendlichen, ausgehend davon, dass sowohl die Schulen, Lehrkräfte und Schülerinnen und Schüler dieser Situation mit unterschiedlichen Ausstattungen sowie unterschiedlichen Fähigkeiten begegneten. Ergebnisse erster explorativer Studien verweisen dabei sowohl auf die Bedeutung digitaler Medien für die Beschulung zuhause (vgl. MPFS 2020; Vodafone Stiftung Deutschland 2020) als auch auf soziale Ungleichheiten in der Verfügung über technische Infrastruktur und über digitale Kompetenzen – und die daraus entstehenden Belastungen (vgl. Anger und Plünnecke 2020; Lochner 2020).

Der Beitrag folgt der Fragestellung, *wie *Kinder und Jugendliche selbst das Gelingen der Fernbeschulung wahrgenommen haben und welche Faktoren dies beeinflussten. Dabei ist ein erster Schritt, aus bestehenden Ansätzen und Forschungsergebnissen ein theoretisches Gesamtmodell des Bedingungsgefüges der Fernbeschulung zu entwerfen. Für die empirischen Analysen des Beitrags ist mit dem so entwickelten Bezugsrahmen von besonderem Interesse, welche Rolle* digitale Medien* während der Schulschließungen spielten. Hierbei war die Annahme leitend, dass die digitalen Medien als infrastrukturelle Voraussetzung des Lernens, als Medium der „Zustellung“ und „Einreichung“ von Lernaufgaben sowie als Mittel der Kommunikation mit Lehrkräften und Mitschülerinnen und -schülern zum Gelingen beigetragen haben.

## Situation und Forschungsstand

Die historisch neue Situation der flächendeckenden Fernbeschulung in Deutschland führte zu völlig neuen Aufgaben für die schulische Praxis bei – vor COVID-19 – insgesamt nur sehr begrenztem Wissen über entsprechende Prozesse der Fernbeschulung und des Homeschoolings (vgl. Kunzman und Gaither 2013; Wissenschaftlicher Dienst des Deutschen Bundestags 2009). Vor der eigenen empirischen Analyse wird daher versucht, mit einer Spurensuche nach entsprechenden Vorarbeiten und Referenzen eine generelle systematische Perspektive zu entwerfen, aus der dann Annahmen für die empirische Analyse abgeleitet werden.

### Fernbeschulung – begriffliche Orientierung

Die Suche nach vergleichbaren Beschulungssituationen, wie jener während der Schulschließungen durch COVID-19 führt zum Ursprung des Konzepts der *distance education* (vgl. Moore et al. 2010), zu historischen Formen des Distanzunterrichts wie den „Schools of the Air“ in Australien, den USA, Kanada und der Sowjetunion (vgl. Nevskaya 2018) sowie der Homeschooling-Bewegung, die bspw. in den USA, Kanada, England und weiteren Ländern in den letzten Jahrzehnten gewachsen ist (vgl. Rothermel 2015). Aber auch wenn es Überschneidungen zwischen Homeschooling und der deutschen Situation während der COVID-19 bedingten Schulschließungen gibt, ist auf einen zentralen Unterschied hinzuweisen: Nach Ray (2017) ist konstituierend für Homeschooling (in seiner ursprünglichen Begriffsverwendung), dass es sich um eine private Form der Bildung handelt, die von Eltern (an)geleitet wird, zu Hause stattfindet und durch eine Loslösung von staatlichen und privaten Schulen sowie deren Rahmenbedingungen und Curricula gekennzeichnet ist. Es ist eine selbstgewählte Praxis, die i. d. R. über einen längeren Zeitraum stattfindet.

Der Term *distance education* bezeichnet dagegen jene Bildungsformate, die institutionell bereitgestellt und instruiert werden, jedoch nicht an einem festgelegten Bildungsort, wie z. B. einer Schule, in Anspruch genommen werden. Entsprechend findet die hier betrachtete Situation der COVID-19 bedingten Schulschließungen eher Parallelen in den erwähnten „Schools of the Air“, die eine Beschulung auch für sehr ländliche und entlegene Gebiete in großen Flächenstaaten ermöglichen (vgl. Stacey 2005) und den amerikanischen „Cyber Charter Schools“. Diese haben sich als digitale Form der Charter Schools^1^ etabliert und fungieren in unterschiedlichen Formen und Angebotsmodi als Alternative zur Präsenzunterrichtung (vgl. Hasler-Waters et al. 2014, S. 379). Wesentlich für diese Art Fernbeschulung ist, dass Inhalte und Struktur der häuslichen Lernaktivitäten nicht von den Eltern, sondern von den Schulen verantwortet werden. Obgleich in diesen etablierten Formen der Fernbeschulung nicht staatliche Sicherheitsauflagen, sondern eine geographische Distanz als konstituierendes Merkmal benannt werden kann, lassen sich deutliche Parallelen zur Situation während der Schulschließungen in Deutschland erkennen. Entsprechend wird für die hier analysierte Unterrichtung in der Zeit der Schulschließungen der Begriff „Fernbeschulung“ verwendet.

In der distance education haben Medien als Vermittler von Lernen über die Distanz schon immer eine tragende Rolle gespielt, von Briefzustellungen im ausgehenden 18. Jh. (vgl. Dieckmann und Zinn 2017, S. 22) (im angloamerikanischen Raum als „correspondence courses“), zu fernsehgestützten Telekollegs und ersten „Distributed Electronic Learning“ Programmen in 1993 (vgl. Horsburgh 2005, S. 22; für Deutschland Dieckmann und Zinn 2017). Mit den angesprochenen technischen Entwicklungen etablierten sich neue Formate der Bildungsvermittlung und Schulformen auf Distanz, wie z. B. Fernstudiengänge oder auch die angesprochenen „Cyber Charter Schools“^2^, die maßgeblich mit den Medien Computer und Internet arbeiten. Diesen Schritt sind auch die australischen „Schools of the Air“, die früher vor allem über das Radio organisiert wurden, gegangen (vgl. Stacey 2005). All diese Formen der Fernbeschulung nutzen mediale Vermittlungsmethoden, mittlerweile zumeist digitale Endgeräte und Internet, wodurch neben synchronen (Live‑)Formaten auch asynchrone oder hybride Bildungsformate möglich sind. So werden zur Gestaltung von Fernbeschulung diverse Applikationen für Online-Präsentationen, Unterrichtseinheiten und kooperative Projektdurchführung genutzt, (individuelle) Interaktionen sind in Chats o. ä. Kommunikationsräumen möglich und Inhalte werden über online-gestützte Lernmanagementsysteme (LMS) didaktisch aufbereitet und vermittelt. Entsprechend ist die Entwicklung des E‑Learnings eng mit diesen Organisationsformen verbunden (vgl. z. B. Ahn 2011, S. 4; Hasler-Waters et al. 2014, S. 380; Schwerdt und Chingos 2015, S. 5). Auch wenn in der Gestaltung der genannten Bildungsformate Lehrkräften die zentrale Rolle zukommt, wird besonders im allgemeinbildenden Bereich die praktische Rolle der Eltern betont, die i. d. R. instruktionale Unterstützung bieten, Motivationsarbeit leisten sowie den Lernprozess und -fortschritt der Schülerinnen und Schüler mit überwachen (vgl. Hasler-Waters 2012, S. 381).

### Situation in Deutschland während COVID-19 bedingter Schulschließungen

Die einzigartigen Umstände der Fernbeschulung ab März 2020 waren Anlass zu wissenschaftlicher Reflexion und politischer Sorge (vgl. Dancer et al. 2020; Sektion Schulpädagogik der DGfE 2020). Neben allen praktischen Problemen wurden vor allem Befürchtungen laut, dass sich bereits bestehende sozioökonomisch bedingte Bildungsungleichheiten durch die Schulschließungen und die Fernbeschulung verstärken (vgl. Fischer et al. 2020). So analysierten bspw. Wößmann et al. (2020) Aussagen von Eltern zum Zeitinvestment von Schülerinnen und Schülern in schulische und nicht-schulische Aktivitäten vor und während der Schulschließungen und stellten dabei eine deutliche Verringerung der schulischen Aktivitäten von durchschnittlich 7,4 zu 3,6 h pro Tag fest (vgl. Wößmann et al. 2020, S. 28). Dabei zeigte sich, dass gerade Nicht-Akademikerkinder und leistungsschwächere Schülerinnen und Schüler überproportional mehr Zeit mit sog. „passiven Aktivitäten“ (Fernsehen, Computer- und Handyspielen sowie sozialen Medien) verbrachten (vgl. Wößmann et al. 2020, S. 38). Ebenso wird in dieser Studie berichtet, dass Nicht-Akademikerkinder häufiger keinen Online-Unterricht und auch seltener individuelle Gespräche mit einer Lehrkraft hatten (vgl. Wößmann et al. 2020, S. 33). Anger et al. (2020b), die Kontaktformen und -häufigkeiten zwischen Lehrpersonen und Lernenden der gymnasialen Oberstufen untersuchten, betonen jedoch, dass gerade ein persönlicher Kontakt zur Schule die schulischen Aktivitäten der Jugendlichen erhöht. Dies bestätigen auch Huber und Helm (2020, S. 249), die einen Zusammenhang zwischen regelmäßigen Kontrollen der Arbeit von Schülerinnen und Schülern und der investierten Lernzeit pro Tag herausstellen.

Zur Praxis der Lehrarrangements während der Schulschließungen entstanden ebenso einige Studien (vgl. auch die Beiträge in diesem Heft), die zunächst vor allem Rahmungen, Prozesse und Bewertungen der Fernbeschulung in den Blick nahmen. Im Zentrum standen dabei auch die häuslichen Ressourcen, die von den Schülerinnen und Schülern eigenständig zu erledigenden „Hausaufgaben“ sowie die Formen des Austausches zwischen Schülerinnen und Schülern und den Lehrkräften. Erste Analysen aus der „FamiLeb“ Studie von Sander et al. (2020), basierend auf Angaben von über 6600 Elternteilen von Schülerinnen und Schülern der Unterstufe in NRW, ergaben, das 95 % der Eltern eine hinreichende Ausstattung lernrelevanter Ressourcen gegeben sahen (vgl. Sander et al. 2020, S. 6) und 60 % der befragten Eltern der Meinung waren, dass für ihre Kinder eine kompetente Unterstützungsperson im Haushalt existierte. Bei der investierten *Lernzeit* zeigte sich, dass Schülerinnen und Schüler in der Mehrheit hinter der, in den Stundentafeln für die jeweilige Schulformen, anvisierten Lernzeit zurückbleiben, d. h. die „Präsenzlehrzeit“ im Schulkontext im heimischen Lernen von mehr als der Hälfte der Lernenden nicht erreicht wird (vgl. Sander et al. 2020, S. 8 f.^3^). Zentrale Kommunikationsformen zwischen Lehrpersonen und Schülerinnen und Schülern waren dabei E‑Mails (83 %) und Lernplattformen (76 %). In der Bewertung der Fernbeschulung äußerten sich die antwortenden Eltern sowohl bzgl. des Kontakts mit verschiedenen Akteursgruppen in der Schule, als auch der Kommunikation, dem bereitgestellten Lernmaterial und den verwendeten digitalen Angeboten überwiegend zufrieden, wobei im Bereich digitaler Angebote die geringste Zufriedenheit vorherrschte (vgl. Sander et al. 2020, S. 15). Dies deckt sich auch mit den Ergebnissen von Wößmann et al. (2020), die herausstellten, dass 57 % der Schülerinnen und Schüler seltener als einmal pro Woche Unterricht im Klassenverbund hatten, die Möglichkeit des Online-Unterrichts von Schulen also „nur vergleichsweise selten genutzt“ wurde (Wößmann et al. 2020, S. 33).

Dazu wurde in der Studie „Schule auf Distanz“ betont (vgl. Vodafone Stiftung Deutschland 2020), dass die große Mehrheit der pädagogischen Fachkräfte die Herausforderung der digital gestützten Fernbeschulung relativ unvorbereitet traf (vgl. Vodafone Stiftung Deutschland 2020, S. 2) – bei Differenzen nach Schulformen, da die Lehrkräfte für Gymnasien (46 %) eine bessere Situation berichteten als jene an anderen weiterführenden Schulen. Hier zeigt sich der bereits vor der Pandemie häufig angesprochener Aufholbedarf der Schulen in Sachen Digitalisierung (vgl. Eickelmann et al. 2019). So wurden vor allem basale Formen digitaler Vermittlung, wie z. B. E‑Mails, in der Fernbeschulung verwendet, während fortgeschrittenere und interaktivere Formen, wie z. B. Clouds oder Lernplattformen, deutlich seltener genutzt wurden (vgl. z. B. MPFS 2020, S. 5; Vodafone Stiftung Deutschland 2020, S. 13 f.; Wößmann et al. 2020, S. 32 f.). Insgesamt sahen jedoch auch in dieser Studie die Lehrkräfte den Zugang zu und den kompetenten Umgang mit digitalen Medien als zentrale Faktoren für gelingende Lernangebote (vgl. Vodafone Stiftung Deutschland 2020). Lehrkräfte an Schulen, welche bereits vor der Schulschließung einen sehr fortgeschrittenen Digitalisierungsprozess durchlaufen hatten, gaben „[…] zu überdurchschnittlich hohen Anteilen (76 %) an, dass die Lernangebote die Schülerinnen und Schüler auch wie von ihnen gewünscht erreichen“ (Vodafone Stiftung Deutschland 2020, S. 23). Probleme in der Realisierung von Lernangeboten werden von Lehrkräften mehrheitlich auf mangelnde technische Ausstattung (75 %) und fehlendes Know-how (53 %) der Lernenden zurückgeführt (vgl. Vodafone Stiftung Deutschland 2020, S. 15). Allerdings stellen sowohl die Sonderauswertung zu PISA 2018 (vgl. OECD 2020, S. 115 ff.) als auch das Schulbarometer (vgl. Huber und Helm 2020, S. 250 ff.) heraus, dass auch den deutschen Schulen, vor allem im Vergleich zu anderen Ländern, technische Ausstattung und dem Lehrpersonal entsprechende Kompetenzen fehlen. Huber und Helm (2020) heben dabei hervor, dass die Daten eine Verbindung der selbsteingeschätzten Kompetenz mit den technischen Ressourcen der Schule offenbaren (vgl. Huber und Helm 2020, S. 252).

Auch Aussagen von Schülerinnen und Schülern zur digitalen Praxis während der Fernbeschulung aus der Sonderbefragung „JIMplus Corona“ unterstützen die Befunde, dass digitale Möglichkeiten für die Fernbeschulung nicht in großem Umfang ausgenutzt wurden. Berichtet wird, dass Schülerinnen und Schüler sich überwiegend auf sich selbst verlassen und Dinge einfach ausprobieren (63 %) oder Unterstützung von ihren Eltern in Anspruch nehmen (32 %), während schulische Instruktionen eher seltener sind (21 %) (vgl. MPFS 2020, S. 1). Gerade die seltenen oder fehlenden Interaktionen mit Lehrkräften oder im Klassenverbund werden vor dem Hintergrund der Relevanz von „classroom management“ und regelmäßigem Feedback für akademische Leistungen von Schülerinnen und Schülern kritisch bewertet (vgl. Huber und Helm 2020, S. 244).

## Theoretischer Analyserahmen

In älteren Theorien zur distance education wurde davon ausgegangen, dass diese theoretisch anders als die klassische Unterrichtssituation konzeptioniert werden müsse. Neuere Ansätze gehen seit den 1990er-Jahren, gerade wegen der zunehmenden Digitalisierung, von einem gemeinsamen theoretischen Rahmen für Unterricht und Fernbeschulung aus (vgl. Simonson et al. 1999), der aber als geschlossenes Modell noch nicht vorliegt. Im Folgenden wird anhand der zusammengetragenen Ergebnisse ein Vorschlag für eine Rahmung entwickelt, die unterschiedliche Dimensionen der (digitalen) Fernbeschulung aufgreift und den Forschungsstand integriert. Von diesem generellen Analyserahmen werden anschließend die Forschungsfragen und Hypothesen für die empirische Studie abgeleitet, die allerdings nicht den Anspruch erhebt, das umfangreiche Modell in Gänze zu testen.

**Figure 1:**
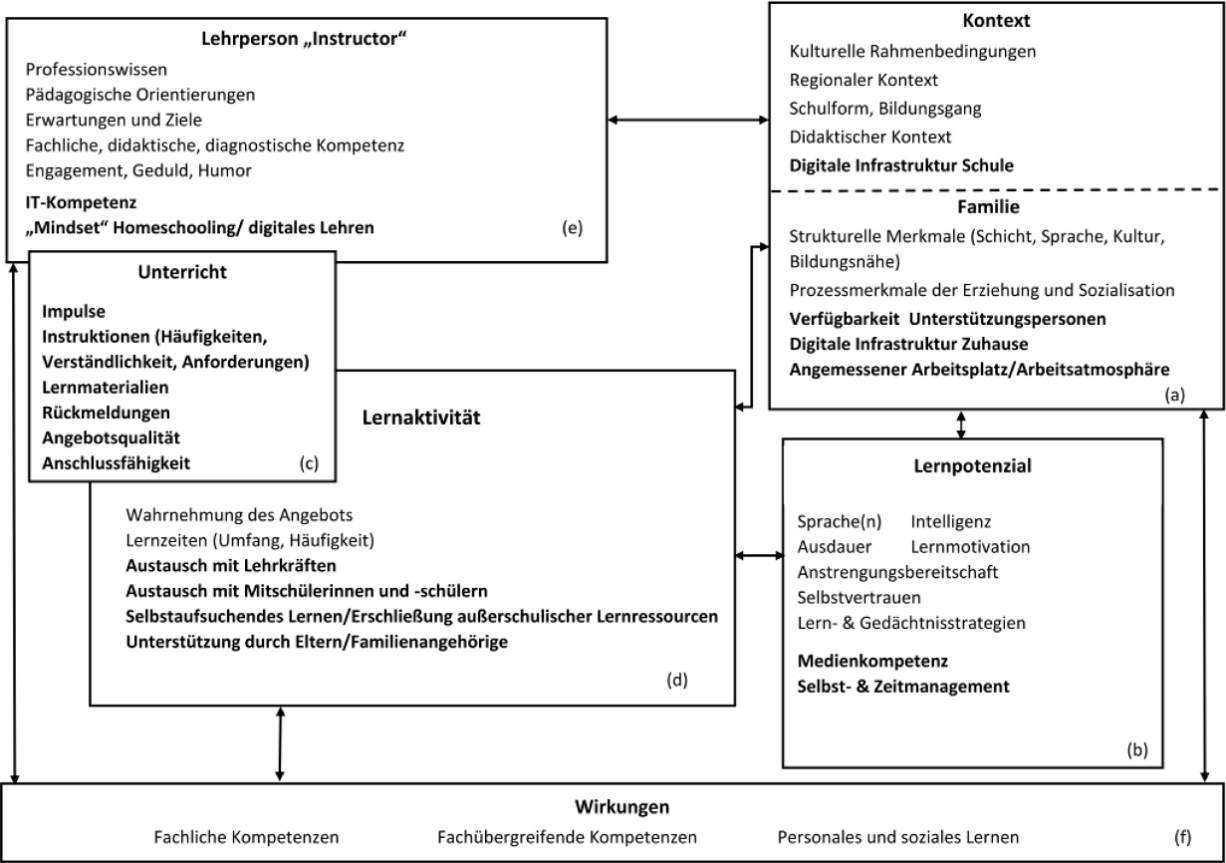

Um der Frage nachzugehen, welche Faktoren den Ablauf von Fernbeschulung bedingen, wird auf das für die Schule entwickelte Angebots-Nutzungs-Modell von Helmke (2012) zurückgegriffen, das den Blick auf Voraussetzungen der Beteiligten, Rahmenbedingungen, Angebotsstrukturen sowie Prozesse weitet und darüber Wirkungen zu erklären sucht. Darin werden Lehrperson, Familie, Kontext, Unterricht, Lernpotential, Lernaktivität sowie Wirkungen als zentrale Dimensionen beschrieben (vgl. Helmke 2012, S. 71). Dieses Modell wird im Folgenden für die Fernbeschulung angepasst und erweitert, zum einen aus dem Befund, dass sich bei der hier in den Fokus genommenen Fernbeschulung die Gewichtung des Lernens vom Unterricht hin zur selbstständigen Einarbeitung und Aufgabenbearbeitung verlagerte und Lernen im Wesentlichen nicht im interaktiven Unterrichtsgeschehen erfolgte. Zum anderen spielten digitale Medien im Fernunterricht eine zentrale Rolle (vgl. Huber und Helm 2020; Vodafone Stiftung Deutschland 2020), weswegen Faktoren für gelingende digitale Lehre ebenfalls einbezogen werden. Die wissenschaftlichen Erkenntnisse zur gelingenden Gestaltung von digitaler distance education, die zumeist aus anderen Kontexten (zu „critical success factors“ von E‑Learning vgl. z. B. Selim 2007) stammen, gilt es, für die aktuelle Situation und neuen Herausforderungen zu „übersetzen“ und nutzbar zu machen (vgl. Huber und Helm 2020, S. 238). Über den Forschungsstand lassen sich daher für die Analyse von Fernbeschulungs-Lernprozessen weitere Einflussfaktoren für ein Angebots-Nutzungs-Modell benennen, die in der Abb. 1 hervorgehoben sind (vgl. Abb. 1).

- *Kontext und Familie*: Helmke (2012) unterscheidet in seinem Modell die Dimensionen Kontext und Familie. Während er mit dem Kontext u. a. kulturelle Rahmenbedingungen, Schulform und Bildungsgang sowie didaktischer Kontext, Klassenzusammenhang und -klima erfasst, werden unter Familie strukturelle Faktoren (Schicht, Sprache, Bildungsnähe) sowie auch Prozessmerkmale der Erziehung und Sozialisation in der Familie subsumiert. Diese beiden Dimensionen sind, so unsere Annahme, in der Fernbeschulung stark verschränkt. Deutlich wird das bei der digitalen Infrastruktur: Auf der Ebene des Kontexts wird für die Fernbeschulung über die von Helmke genannten Faktoren hinaus die digitale Lerninfrastruktur, die die Schulen bereitstellen, wie z. B. die Lernplattformen, angesehen (vgl. Vodafone Stiftung Deutschland 2020), für deren Nutzung aber auch eine entsprechende familiäre Medienausstattung benötigt wird. „Information Technology“ betont auch die Forschung zum E‑Learning als zentrale Dimension, zu der u. a. Nutzerfreundlichkeit, Zugangs- und Nutzungsmöglichkeiten und das interaktive Potenzial der eingesetzten digitalen Medien zählen (vgl. u. a. Benigno und Trentin 2000, S. 8; Selim 2007, S. 407; Soong et al. 2001, S. 119; Volery und Lord 2000, S. 220). Mit Blick auf die häusliche/familiale Lernumgebung wird die digitale Infrastruktur, die Verfügung über angemessene Arbeitsplätze sowie das Vorhandensein von Unterstützungspersonen und ein förderliches Lernklima als bedeutsam angenommen (vgl. Sander et al. 2020). Auch die Rahmung des Lernprozesses findet trotz weiter existierenden Klassenstruktur wesentlich in der Familie statt (vgl. Lochner 2020, S. 7; Sander et al. 2020, S. 10), sodass der Ressource Eltern (im Sinne der Anwesenheit zur Unterstützung als auch deren Befähigung zur Unterstützung und Hilfe) eine größere Bedeutung im Lernprozess zukam (vgl. z. B. Huber und Helm 2020, S. 244 ff.). Entsprechend tritt zu den oben benannten Einflussfaktoren *Unterstützungszeit durch Familienmitglieder und deren Befähigung*, die in einem Wechselverhältnis stehen, hinzu (vgl. auch Dietrich et al. 2020).
- *Lernpotenzial*/*persönliche Voraussetzungen*: Unter den von Helmke (2012) genannten Aspekte lassen sich über den Forschungsstand zur Fernbeschulung besonders bedeutsame Faktoren wie die Fähigkeit zum selbstregulierten Lernen, eigene Strukturiertheit, eigene Lernzielorientierung sowie Selbstvertrauen betonen (vgl. Huber und Helm 2020; Soong et al. 2001),– mit einer möglicherweise noch stärkeren Bedeutung für die Eigenarbeit zu Hause im Vergleich zum Lernen im Schulunterricht. Auch für die individuellen Fähigkeiten im Umgang mit digitalen Medien wird ein Einfluss auf das Gelingen von entsprechenden Lernprozessen angenommen (vgl. bspw. Pächter et al. 2010; Selim 2007, S. 406). Benigno und Trentin (2000, S. 4 ff.) führen im E‑Learning-Diskurs „Student characteristics“ als wichtige Kategorie in der Analyse von „critical success factors“ an, worunter sie vor allem den Wissensstand zur vermittelten Thematik, die bisherigen Erfahrungen mit Fern- und Onlinelehre, Konditionen der Lernumgebung und das technische Know-how fassen. Ebenso fassen Soong et al. (2001, S. 119) unter „human factors“, zu denen sie auch investierte Lernzeit zählen, die technische Kompetenz und ein „constructive mindset“ in Bezug auf Lernen, sowohl für Lernende als auch Lehrende, als „CSF“ auf. Selim (2007, S. 409 ff.) betont darüber hinaus die Bedeutung von Motivation und Selbstverpflichtung der Lernenden als elementar für den Erfolg und ergänzt auch die Punkte Zeitmanagement und Disziplin.
- Unterricht und Lernaktivitäten sind bei Helmke (2012) zwei eigenständige Dimensionen, die für Fernbeschulung möglicherweise anders konzeptioniert werden müssen. So scheint – solange Fernbeschulung vor allem eigenständige Aufgabenbearbeitung bedeutet – die Dimension *Unterricht* über die Analyse möglicherweise vorhandener digitaler Unterrichtsformen primär über den Blick auf Lernaufgaben und -rückmeldungen sowie ihrer Qualität abzubilden zu sein. Dabei ist anzunehmen, dass die konkreten Instruktionen und die Lernmaterialien, aber auch Lernkontrolle und Feedback für das Gelingen besondere Bedeutung erlangen. Bezogen auf Kriterien der Prozessqualität, wie sie in den Basisdimensionen guten Unterrichts (strukturierte Unterrichtsführung, schülerorientiertes Sozialklima sowie kognitive Aktivierung) (vgl. Klieme und Rakoczy 2008, S. 228) formuliert sind, ist die Frage, wie diese auf digitalen Wegen wirksam sind – und es bleibt die Frage, wieweit diese Interaktionen(z. B. über Videounterricht) voraussetzen.
- Bei den *Lernaktivitäten* ist der Einfluss der *Prozesse eigenständigen und interaktiven Lernens* zu prüfen – über den Umfang der Lernzeiten, Vorhandensein direkten Austausches mit Lehrkräften und/oder Mitschülerinnen und Mitschülern, Lernprozesse mit Eltern/Familienangehörigen sowie eigenständiger digitaler Lernaktivitäten.^4^ Die Unterstützung in Lernprozessen durch Eltern/Familienangehörige wird hierbei bewusst der Dimension Lernaktivität zugeordnet und nicht der des Unterrichts, da hier von keinem regelhaften, systematischen, strukturell von der Schule gesteuerten Prozess ausgegangen wird, sondern je nach Elternhaus unterschiedlich und vor allem erfahrungsbasiert erfolgt. Die benannten Faktoren erwiesen sich auch in der Forschung zum E‑Learning im Hochschulkontext als relevant. Benigno und Trentin (2000) benennen in einer Rahmung zur Evaluation von E‑Learning sowohl Lernmaterialen als auch die Interaktion mit Peers und die Lernumgebung als wichtige Untersuchungspunkte. Selim (2007, S. 406) und Soong et al. (2001, S. 119) arbeiten ebenfalls das Level an möglicher Kollaboration im Lehr‑/Lernsetting als wichtigen Faktor heraus. Auch Govindasamy (2002, S. 289 ff.) diskutiert als Erfolgsfaktoren von E‑Learning die Struktur und Entwicklung eines Kurses bzw. Lehrangebots, da die Herausforderung darin liegt, etablierte Strukturen von Präsenzlehre funktional in digitale Lernumgebungen zu übertragen. Dabei spielt die Lehrperson, die die entsprechende Planung und Umsetzung verantwortet, eine zentrale Rolle.
- *Lehrperson*: Für die Dimension Lehrperson sind über die Faktoren des klassischen Angebots-Nutzungs-Modell hinaus mit Blick auf den Forschungsstand Faktoren wie IT-Kompetenz, sowie Haltung und „Mindset“ mit Blick auf Fernbeschulung generell und digitale Lehrangebote im Besonderen als bedeutsam anzunehmen. Gestützt wird dies auch aus dem E‑Learning-Diskurs in anderen Lehrkontexten: Als personale Voraussetzungen spielen die digitalen Kompetenzen sowie die Einstellung zur digitalen Lehre und den Lernenden eine wichtige Rolle (vgl. Baylor und Ritchie 2002; Selim 2007, S. 404 f.; Soong et al. 2001, S. 108 ff.; Volery und Lord 2000, S. 220 ff.). Dabei ist besonders auch der Lehrstil bei digitalen „Live“-formaten noch eine offene Frage, auch wenn dies im März/April 2020 eher selten praktiziert wurde. Auch die angebotene Beratung und Unterstützung ist ein bedeutender Faktor für das Gelingen von digitaler Fernbeschulung. In den bereits durchgeführten Studien zur Situation in Deutschland wurden dabei Vermittlungs- und Kontaktkanäle sowie -formen und -häufigkeiten zwischen Lehrenden und Lernenden betont (vgl. Anger et al. 2020; Dietrich et al. 2020; Wößmann et al. 2020).
- *Wirkungen*: Auf der Ebene der (intendierten) Wirkungen gibt es wenig Argumente, von den von Helmke beschrieben Dimension abzuweichen. Allerdings ist eine offene Frage, ob die sogenannten „erzieherischen Wirkungen der Schule“ (Helmke 2012, S. 71) in Fernbeschulung eine aktive Rolle spielen und Gegenstand der schulischen Vermittlung sind oder sich während der Fernbeschulung die Ausrichtung der Lehre stärker auf fachliche und (schulbezogene) fachübergreifende Kompetenzen konzentriert. Mit Blick auf die Wirkungen von digitaler/virtueller Beschulung auf fachliche Kompetenzen stellen Schwerdt und Chingos (2015, S. 14) in ihrer Untersuchung fest, dass Testscores von Schülerinnen und Schülern, die virtuelle Kurse in Algebra I und Englisch I belegten, mindestens genauso gut ausfielen wie die jener, die entsprechende Kurse analog in der Schule besuchten. Mehr oder weniger intendierte (Neben)Wirkungen des Fernunterrichts dürften zudem der Erwerb von Kompetenzen im Umgang mit Computern, Internet und Lernplattformen sein (vgl. Schaumburg et al. 2019) sowie durch die Praxis verbesserte Fähigkeiten zum selbstregulierten/selbstgesteuerten Lernen (vgl. Otto et al. 2011), wobei diese beiden Faktoren sowohl Voraussetzungen als auch Nebenwirkungen der Fernbeschulung sind bzw. sein können.

Das entworfene Modell ist als ein genereller und umfassender Ansatz gedacht, um das Bedingungsgefüge der Fernbeschulung angemessen erfassen zu können. Die eigene Studie zielt nicht auf eine Modellierung des gesamten Rahmens, sondern nutzt diesen als theoretische Grundlage zur empirischen Prüfung – anhand der Perspektive von Schülerinnen und Schülern – folgender Annahmen:

- dass sowohl die häufigere Anwesenheit von Eltern sowie eine gute Medienausstattung zu Hause das Gelingen der Fernbeschulung positiv beeinflussen (Dimension a),
- dass Kinder aus nichtakademischen Elternhäusern aufgrund der beschriebenen Einflussfaktoren verstärkt Schwierigkeiten mit der Fernbeschulung haben (auch Dimension a),
- dass vor den Schulschließungen vorhandene individuelle Fähigkeiten im Umgang mit digitalen Medien einen Einfluss auf das Lernen zu Hause besitzen (Dimension b),
- dass der Einsatz digitaler Medien und insbesondere interaktiver digitaler Kommunikations- und Vermittlungsformen sich positiv auf das Lernen zu Hause auswirken (Dimension c) sowie
- dass ein größerer Umfang von Lernzeiten zu Hause einen positiven Einfluss auf gelingendes Lernen hat (Dimension d).

## Daten und Methoden

Die für die folgenden Analysen verwendeten Daten entstammen einer quantitativen Online-Studie aus dem Zeitraum Mai–Juni 2020 mit Schülerinnen und Schülern aus weiterführenden allgemeinbildenden Schulen in den Bundesländern NRW und Rheinland-Pfalz. Im Mittelpunkt der Befragung stand die Lernpraxis der Schülerinnen und Schüler während der Fernbeschulung mit besonderem Fokus auf der Rolle digitaler Medien. Die Befragung entstand im Kontext des vom BMBF geförderten Forschungsprojektes „Ganztag digital“, welches den Einsatz von digitalen Medien im Kontext von Ganztagsschulen untersucht (www.ganztag-digital.de).

### Studiendesign und Stichprobe

Die Befragung wurde als online-gestützte Querschnittsbefragung von Schülerinnen und Schülern der Klassen 5–12 mit dem Tool SoSci-Survey durchgeführt, und konnte via Computer, Tablet oder Smartphone beantwortet werden. Teilnehmende wurden maßgeblich durch die am Forschungsprojekt teilnehmenden Schulen aus NRW gewonnen. Einbezogen wurden Schülerinnen und Schüler von acht Schulen, sechs davon waren Gymnasien. Kontaktiert wurden die Teilnehmenden über ein Anschreiben der Schulen an die Eltern, die wiederum ihre Kinder zur Teilnahme an der Umfrage ermutigten und den entsprechenden Link weiterleiteten. Insgesamt lagen für 1128 Schülerinnen und Schüler vollständig ausgefüllte Fragebögen vor, der Rücklauf lag zwischen 20 und 45 % der Schülerinnen und Schüler der jeweiligen Schule. Verabredungsgemäß erhielten die einzelnen Schulen im Juni 2020 Berichte mit schulspezifischen Ergebnissen.

Für die Stichprobe ist über einen Ganztags- und einen Gymnasialbias hinaus von mehrfachen Selektionsprozessen auszugehen: der Bereitschaft zur Teilnahme der Schulen an einer Studie, der Bereitschaft der Eltern zur Weitergabe und Unterstützung der Befragung sowie der tatsächlichen Teilnahme der Kinder und Jugendlichen an der Befragung. Über die Auswahl der Schulen und diese Selektionsprozesse sind die Daten nicht systematisch auf eine Grundgesamtheit zurückzuführen, sodass auf die Verwendung des Signifikanzprinzips verzichtet wird. Insgesamt wurden zu 87 % Gymnasiastinnen und Gymnasiasten erreicht, 49 % der Antwortenden haben mindestens ein akademisch qualifiziertes Elternteil (vgl. Tab. 1). Offen bleibt, inwieweit eine Nichtteilnahme nach Einladung über die Eltern ein Kommunikationsproblem oder Ausdruck fehlenden Interesses war oder ob Schülerinnen und Schüler aufgrund mangelnder Medienausstattung oder -kompetenz nicht an der Umfrage teilgenommen haben., was als eine der Limitationen der Studie betont werden muss.

**Table 1:** 

Variable	Ausprägungen		
*–*	*–*	**In %**	**Abs**
Geschlecht	Weiblich	58,9	653
	Männlich	40,6	450
Überwiegend im Haushalt gesprochene Sprache	Deutsch	86,5	960
	Andere Sprache	13,5	150
Akademisches Elternhaus	Kein akad. Elternteil	51,5	528
	Min. 1 akad. Elternteil	48,5	497
Wohnsituation	In Wohnung	40,5	449
	In Einfamilienhaus	55,0	610
	Sonstiges	4,5	50
Schulform	Gymnasium	87,4	970
	Andere Schulformen Sek I	12,6	140
Klassenstufen (zusammengefasst)	Stufen 5–7	47,3	530
	Stufen 8–10	38,5	434
	Stufen 11–12	13,9	156
*–*	*–*	**MW**	**SD**
Alter	In Jahren	13,5	2,95

*n* = 1128

Die Teilnehmenden waren im Schnitt 13,5 Jahre alt, dabei handelte es sich zu 58 % um weibliche Personen. 47 % der Antwortenden besuchten zum Befragungszeitraum die Klassen fünf bis sieben, 39 % die achten bis zehnten Klassen und 14 % der Antwortenden stammten aus den Stufen elf und zwölf. Zum Zeitpunkt der Befragung lebten 56 % der antwortenden Schülerinnen und Schüler in Einfamilienhäusern und 39,5 % in Wohnungen, als überwiegend im Haushalt gesprochene Sprache wurde Deutsch (87 %) angegeben.

### Operationalisierung der Variablen und Verteilungen in der Stichprobe

#### Abhängige Variable

Da aus den Aussagen der Kinder und Jugendlichen keine Wirkungen abgeleitet werden können, rückt das „Gelingen“ des eigenständigen Lernens während der Fernbeschulung an die Stelle der zentralen abhängigen Variablen. In einem explorativen Vorgehen werden dabei die Selbsteinschätzungen der Schülerinnen und Schüler zur Bewältigung der Herausforderungen zusammengefasst: Zur Operationalisierung des Modells wurde ein Konstrukt verwendet, dass vier Items mit dichotomen Antwortformat zu Fragen umfasst, wie den Schülerinnen und Schülern das Lernen zu Hause gelingt (vgl. Tab. 2, Items teilweise invertiert).

**Table 2:** 

Items	Zustimmung (in %)	Itemschwierigkeit	Trennschärfe
Aufgaben sind zu schwer (für IRT-Analyse invertiert)	17,7	−1,09	2,95
Aufgaben kann ich in der Mehrheit gut verstehen	82,2	−1,10	3,33
Aufgaben kann ich in der Regel ohne Hilfe lösen	80,3	−1,21	1,72
Ich bin mit der Aufgabenmenge überfordert (für IRT-Analyse invertiert)	37,8	−0,42	1,94

*n* = 1110

Ausprägungen nein = 0/ ja = 1

Test auf Eindimensionalität nach Drasgow and Lissak *p* = 0,129

Diese Items können als selbstwahrgenommene Fähigkeit zur Bewältigung der Anforderungen oder auch als subjektiv wahrgenommene Schwierigkeiten mit den Anforderungen der Fernbeschulung interpretiert werden – in den Augen der Autor*innen sind sie in beiden Lesarten ein Indikator für das Verhältnis zwischen der subjektiven Einschätzung sowohl der Aufgabenanforderungen als auch der eigenen Fähigkeiten/Möglichkeiten.

Für einen Indikator, der diese Informationen zusammenfasst, wurden mit den dichotomen Variablen, sowohl IRT-Modelle (Rasch-Modell) als auch das Birnbaummodell (mit R, Pakete ltm) geschätzt. Aufgrund des Modellvergleichs wurde dem Birnbaum-(2-Pl‑)Modell der Vorzug gegeben. Die geschätzten Personenwerte (factor scores) zur Einschätzung haben einen Mittelwert von −0,12 (Min −1,65; Max 0,45) und eine Standardabweichung von 0,68 (*n* = 1115).

#### Unabhängige Variablen

Die Darstellung der unabhängigen Variablen erfolgt anhand des unter Punkt 3 skizzierten Rahmenmodells, auch wenn nicht alle Dimensionen mit den vorliegenden Daten vollständig operationalisiert werden können.

- *Kontext und Familie: *Für die Prüfung des Einflusses schulischer Faktoren wurde die jeweilige Klassenstufe metrisch erfasst (von 5–12). Das Vorhandensein einer schulischen Lernplattform wurde als binär codierte Variable erhoben, 75 % der Befragten gaben die Existenz einer schuleigenen digitalen Lernplattform an. Der Klassenzusammenhang wurde aus Datenschutzgründen nicht erhoben, so wurde die Mehrebenen-Struktur der Daten (Schülerinnen und Schüler in 8 Schulen) über Dummy-Variablen für die Einzelschulen (also als feste Effekte für die jeweilige Schule) modelliert. Aufgrund begrenzter Effekte wurde im Endmodell eine binär codierte Variable für die Schulform Gymnasium (1 = ja) verwendet.
  Zur *familiären Herkunft* wurden die Informationen zur überwiegend im Haushalt gesprochenen Sprache (Deutsch = 0/ eine andere Sprache = 1) und zum Bildungsstatus der Eltern (mindestens 1 Elternteil mit akadem. Abschluss = 1) als binär codierte Variablen verwendet (vgl. Tab. 1). Zur Erhebung der Verfügbarkeit digitaler Infrastruktur zu Hause wurde gefragt, inwieweit die Schülerinnen und Schüler Internetzugang sowie Computer, Laptop, Drucker und Scanner sowie Smartphone zur Verfügung hatten. Sowohl beim Internetzugang (98 %) als auch Smartphone-Besitz (97 %) kann von einer Vollabdeckung gesprochen werden; 90 % der Befragten gaben an, einen Computer/Laptop nutzen zu können. Diese Daten entsprechen auch den Ergebnissen der vorgestellten Studien, die ebenfalls von einer ausreichenden technischen Ausstattung berichten (vgl. z. B. MPFS 2020; Sander et al. 2020). Für die Analysen wurde nur der Zugang zu Computer/Laptop als binäre Variable verwendet. Als *häusliche Ressource* wurde auch die Anwesenheit mindestens eines Elternteils zu Hause (s. oben) erfasst. Zum Befragungszeitpunkt war bei 29 % der Befragten mindestens ein Elternteil vollständig zu Hause (inkl. Alleinerziehende), bei 14 % beide Elternteile.^5^ Für die Modellierung wurde eine binäre Variable, „mindestens ein Elternteil voll zu Hause“, verwendet. Zudem wurde erhoben, ob die Befragten regelmäßig Unterstützung von Eltern/Familienmitgliedern erhielten.
- *Lernpotenziale: *Neben den schon in der Stichprobenbeschreibung enthaltenden Personeninformationen zu Geschlecht und Klassenstufe wurde eine Variable verwendet, die die schulische Leistungsfähigkeit widerspiegeln soll und aus dem arithmetischen Mittel der letzten Schulnoten in Mathematik, Deutsch und erster Fremdsprache gebildet wurde (MW 2,60; SD 0,83). Als Maß für die individuellen Computerfähigkeiten, wurde aus fünf Items zu Fähigkeiten, die die Befragten im Umgang mit Computer und Internet vor Beginn der Schulschließungen angaben, ein IRT-skaliertes Maß (wiederum über ein Birnbaum-Modell) gebildet (vgl. Tab. 3).
  Die für das Modell geschätzten Personenwerte (factor scores), haben einen Mittelwert von −0,03 (Min −1,41; Max 1,01) und eine Standardabweichung von 0,74 (*n* = 1113).
  Maße zur individuellen Lernmotivation, zur Lernzielorientierung oder auch zur eigenen Strukturiertheit im Lernen wurden in der Studie leider nicht erhoben.
- *Unterricht und Lernaktivitäten: *Zentral für die Erfassung der Lernprozesse und Aktivitäten war die in Tab. 4 dargestellte Abfrage, zum Ablauf der Aufgabenbearbeitung und des Lernens.
  Sichtbar wird, dass längst nicht alle Befragten bearbeitete Aufgaben regelmäßig wieder bei der Schule einreichen mussten, und auch, dass nur 20 % der Befragten von regelmäßigen Rückmeldungen zu den Leistungen berichteten (bei 21 %, die angaben, keine Rückmeldungen bekommen zu haben). Bezogen auf den Austausch wird auch deutlich, dass nur eine Minderheit der Befragten Videounterricht (24 % ab und zu und regelmäßig, 18 % regelmäßig) oder direkten Kontakt mit Lehrenden per Video (22 % ab und zu, 10 % regelmäßig) hatten. Hier zeigen sich Parallelen zu den in Abschn. 1.2 beschriebenen Studien (vgl. z. B. MPFS 2020; Vodafone Stiftung Deutschland 2020; Wößmann et al. 2020). Der digitale Austausch über Schulaufgaben unter den Schülerinnen und Schülern fand dagegen deutlich häufiger statt.
  Für die Modellierung wurden jeweils Variablen mit den Ausprägungen 1 = „ja, regelmäßig“/ 0 = „nein bzw. ab und zu“ zu den Items verwendet. Die beiden Items zu helfenden Personen wurden in eine binär codierte Variable (im Sinne von regelmäßiger Hilfe durch Eltern oder andere Personen, von der insgesamt 26 % der Befragten berichteten) zusammengefasst. Einzelitems sind für eine entsprechenden Modellierung unbefriedigend, da bei den Einzelitems die Reliabilität der Messungen nicht kontrolliert werden kann und stellen hier eine Notlösung dar, da lagen zu den verschiedenen relevanten Aspekten leider keine weiteren Informationen erhoben wurden.
  *Lernzeit pro Tag*: Abgefragt wurde, wie viele Stunden pro Tag die Befragten in der letzten Woche zu Hause für die Schule gearbeitet haben. Für die Gesamtstichprobe ergab sich ein arithmetisches Mittel von 3,6 h (SD 2,3 h). Diese Daten stehen in Einklang mit den vorgestellten Ergebnissen anderer Studien, wie bspw. Wößmann et al. (2020, S. 28), die ebenso von 3,6 h pro Tag sprechen.
- *Lehrperson: *Zur jeweiligen Lehrperson wurden leider keine Angaben erhoben, obwohl die sehr unterschiedlichen Bewertungen beim Lernen nach Fächern auf spezifische Unterschiede hinweisen könnten. So können Art der Aufgabeneinforderung, Rückmeldungen und Videounterricht (s. oben) nur ansatzweise als Hinweise auf den Lehrstil verstanden werden.

**Table 3:** 

Items	Zustimmung (in %)	Itemschwierigkeit	Trennschärfe
Insgesamt mit einem Computer umgehen	60,1	−0,32	2,16
Texte mit Office Programmen wie z. B. Word schreiben und bearbeiten	52,6	−0,08	2,44
E‑Mails schreiben	54,4	−0,15	1,72
Lernplattform benutzen	29,5	1,04	1,01
Im Internet zu recherchieren	82,9	−1,29	1,90

*n* = 1110

Ausprägung ‚konnte ich noch nicht gut‘ = 0/ ‚konnte ich schon gut‘ = 1

Test auf Eindimensionalität nach Drasgow und Lissak *p* = 0,248

**Table 4:** 

Items	Nein (in %)	Ja, zum Teil/ab und zu (in %)	Ja, regelmäßig (in %)
Müsst ihr eure Schularbeiten online einreichen/abgeben?	6,8	54,0	3,29
Bekommt ihr eine Rückmeldung zu den einzelnen Leistungen von euren Lehrern und Lehrerinnen?	21,0	58,6	20,4
Macht ihr auch Unterricht per Videokonferenz?	58,3	23,8	17,8
Tauscht ihr euch auch untereinander ohne Lehrer und Lehrerinnen über die Schularbeiten aus (mit Whats App etc.)	24,7	42,1	33,2
Helfen dir deine Eltern bei den Schularbeiten?	35,7	41,6	22,7
Helfen dir andere Personen bei den Schularbeiten (z. B. Großeltern. Geschwister)?	64,9	26,5	8,5

*n* = 1110

### Auswertungsmethoden

Um die Analysen in Ansätzen entlang des erweiterten Analysemodells durchführen und auch Wechselwirkungen zwischen den Dimensionen berücksichtigen zu können, wurde als multivariate Analyse ein Pfadmodell geschätzt. Dies bedeutet auch eine Komplexitätsreduktion, sowie eine Zusammenfassung vorher differenzierter erhobener Variablen. Mit dem Pfadmodell wird keinen kausalen Wirkungen, sondern der Frage nachgegangen, ob sich die vermuteten Beziehungen in den empirischen Daten zeigen lassen. Geschätzt wurde das Pfadmodell mit dem R Paket lavaan mit einem „diagonally weighted least squares“-Schätzer (DWLS), der wie ein Full-Information-Likelihood-Schätzer die Informationen aller verfügbaren Fälle aufnimmt und Fälle nicht wegen einzelner fehlender Werte ausschließt. In der Grafik zum Pfadmodell sind alle modellierten Pfade mit standardisierten Koeffizenten abgetragen. Als Maß für die im Text berichtete *Bedeutsamkeit* von Pfaden wird Cohens Effektgröße f^2^ verwendet, die Chin (1998) für Pfadmodelle empfiehlt. Dabei wird das Modell jeweils einmal mit und ohne die jeweilige Variable geschätzt, das berechnete f^2^ wird bei Werten > 0,02 als kleiner Effekt und bei Werten > 0,15 als mittlere Effekt interpretiert.

Wie schon beschrieben, wurde für eine Erfassung der Fähigkeit der Schülerinnen und Schüler, ihre schulischen Aufgaben und Anforderungen zu Hause zu bewältigen, zudem eine IRT-Skalierung (R Paket ltm) vorgenommen, um eine entsprechende abhängige Variable als Annäherung an eine von den Schülerinnen und Schülern wahrgenommene erfolgreiche Bewältigung der Anforderung der Fernbeschulung verwenden zu können.

## Analysen

Mit Blick auf die formulierten Annahmen wurde ein Pfadmodell anhand des postulierten Rahmenmodells geschätzt, das als zentrale abhängige Variable die geschätzten Faktorwerte des Raschmodells zur selbstwahrgenommenen Möglichkeit zur Bewältigung der Lernaufgaben/-anforderungen der Fernbeschulung abbildet. Im Kern basiert das auf vier Regressionengleichungen, die sich aus dem formulierten Rahmen ergeben. Neben der benannten Variable wurden in den Gleichungen des Pfadmodelles als abhängige Variablen der Umfang der Lernzeit, die selbsteingeschätzte Computerfähigkeiten sowie das Vorhandensein von Hilfspersonen im Haushalt aufgenommen.

**Figure 2:**
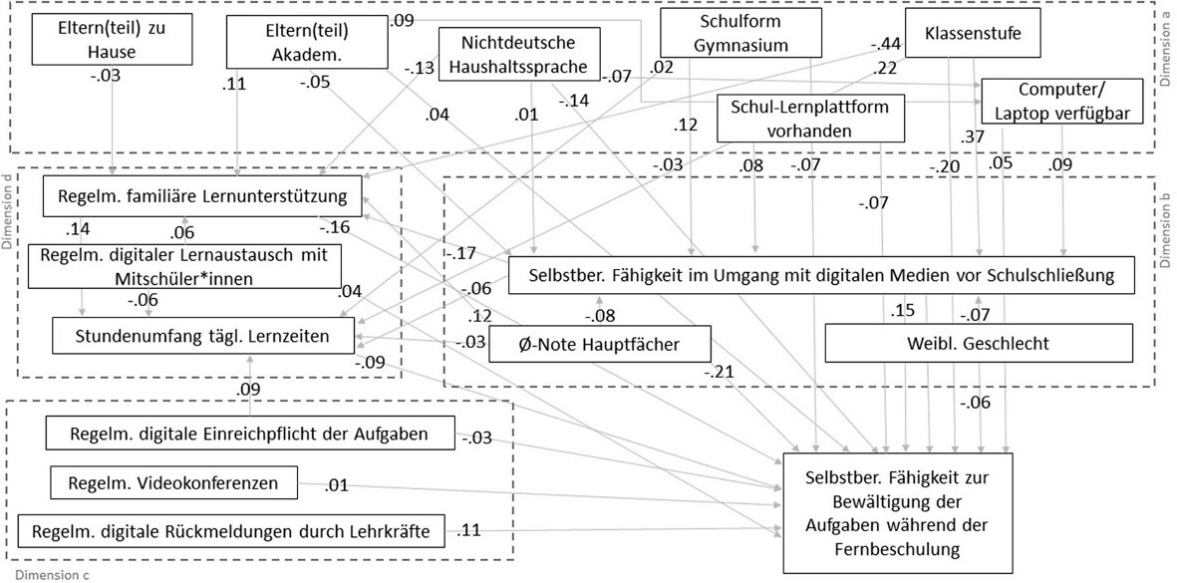

Vor der Modellierung wurde geprüft, inwieweit sich die Fähigkeit zur Bewältigung der Aufgaben (und auch die Computerfähigkeiten) zwischen den Schulen systematisch unterscheidet. Bei Varianzzerlegung bzw. Ermittlung der Effektstärke für die Schulvariable ergab sich ein Varianzanteil von nur 3 % auf Schulebene bzw. ein eta^2^ von 0,03, so dass auf eine explizite Modellierung des Schulkontextes durch fixe oder zufällige Effekte verzichtet und stattdessen auf Schulebene nur die Variable zur Schulform eingebracht wurde. Die Modellfitwerte des geschätzten Gesamtmodells weisen insgesamt eine gute Passung der Daten auf das Modell aus (vgl. Abb. 2), so dass insgesamt von einer Erklärungskraft des Modells ausgegangen werden kann.^6^

Mit Blick auf die formulierten Hypothesen lassen sich aus allen Dimensionen Einflüsse auf die subjektiv wahrgenommene Bewältigung der Aufgaben während der Fernbeschulung annehmen, gleichzeitig werden nicht alle konkret formulierten Annahmen durch das Modell bestätigt. In der Frage der Bedeutung des Digitalen zeigen sich erwartungsgemäß Zusammenhänge für die Dimension *Unterricht* darin, dass sich digitale Rückmeldungen positiv auf das Lernen zu Hause auswirken (f^2^ > 0,2). Die Existenz von regelmäßigen Videokonferenzen hat für diese Stichprobe dagegen bspw. kaum einen Effekt auf erfolgreiche Aufgabenbewältigung zu Hause. Möglicherweise waren in der Perspektive der Schülerinnen und Schüler die schulischen Hausaufgaben und Videokonferenzen unterschiedliche bzw. wenig verbundene Elemente, oder vielleicht wurden in den (wenigen) Videokonferenzen andere Dimensionen, z. B. Organisatorisches, verhandelt als in der inhaltlichen Arbeit in den Hausaufgaben, für die die Lehrkräfterückmeldungen bedeutsamer waren. Aus der Dimension *Lernpotenziale* zeigten sich insbesondere die vor den Schulschließungen vorhandenen individuellen Fähigkeiten im Umgang mit digitalen Medien und die bisherigen Schulnoten als positiv bedeutsam für die gelingende Aufgabenbewältigung (jeweils f^2^ > 0,2). Diese Ergebnisse entsprechen den beschriebenen Forschungsergebnissen aus dem E‑Learning-Diskurs und weisen auf die Relevanz eines geschulten Umgangs mit digitalen Medien für gelingende Fernbeschulung hin. Die negativen Pfadkoeffizienten, die eine eher geringere Selbsteinschätzungen von Mädchen in den Computerfähigkeiten und der Bewältigung der Hausaufgaben signalisieren, sollten aufgrund der geringen Effektstärken nicht überbewertet und vor allem als Auftrag zu weiterer Forschung verstanden werden.

Mit Blick auf *den Kontext und den familiären Hintergrund* weist eine nicht-deutsche Haushaltssprache einen negativen Zusammenhang zur berichteten Fähigkeit auf, die Hausaufgaben zu bewältigen, während der direkte Effekt für den Bildungsstand der Eltern nur gering ist. Über Bildungsstand und Haushaltssprache ergeben sich aber auch indirekte Herkunftseffekte, da diese Faktoren mit dem Vorhandensein eines Computers/Laptops zu Hause korrelieren. Dies wiederum beeinflusst die Bewältigungsmöglichkeit der Aufgabenbearbeitung kaum direkt, aber indirekt über den Einfluss auf die Computerfähigkeiten der Schülerinnen und Schüler. Diese indirekte Logik zeigt sich auch – bei allerdings sehr geringer Effektstärke – beim Einfluss von Lernplattformen: wenn Schulen Lernplattformen haben, hat dies einen positiven Einfluss auf die selbstberichteten Computerfähigkeiten (vgl. auch Huber und Helm 2020), während der direkte Effekt von Lernplattformen auf die Hausaufgabebewältigung sogar negativ ausfiel. Dies könnte dahingehend gedeutet werden, dass kurzfristig neu etablierte/eingesetzte Lernplattformen die Aufgabenbewältigung für Schülerinnen und Schüler eventuell verkomplizierten. Hier wäre weitere Forschung nötig, um den Hintergrund dieses Effektes aufzuklären.

Bei den *Lernaktivitäten* zeigt sich nur ein sehr schwacher Einfluss des Austausches unter den Schülerinnen und Schülern darauf, wie gut diese die Aufgaben bewältigen konnten. Der Umfang an Lernzeiten zu Hause steigt vor allem mit der Pflicht zur Einreichung von Aufgabenergebnissen, einer intensiven elterlichen Unterstützung und ist in höheren Klassenstufen größer als in den unteren. Er ist, entgegen der formulierten Annahmen, negativ mit einer subjektiv gut eingeschätzten Bewältigung der Aufgaben assoziiert, ebenso wie regelmäßige elterliche Unterstützung (die wiederum von Sprachvermögen und Bildungsstand abhängig ist).^7^

In der Gesamtschau der Ergebnisse kann zunächst bilanziert werden, dass das postulierte Modell zumindest über die hier empirisch abbildbaren Bereiche als Gesamtmodell einen zufriedenstellenden Fit erreicht, auch wenn die Einzeleffekte an vielen Stellen eher klein sind (und auf eine inferenzstatistische Absicherung verzichtet werden musste). Die, ebenfalls in anderen Studien (vgl. Abschn. 1.2) herausgearbeiteten Verbindungen zwischen der erfolgreichen Bewältigung von (Aufgaben in der) Fernbeschulung und herkunftsbedingten Voraussetzungen (Bildungsstand und Sprache der Eltern) sowie individuellen Fähigkeiten (vorhandene Computerfähigkeiten, Schulerfolg) wurden auch in dieser Stichprobe deutlich.

Bzgl. der digitalen Kommunikation deuten die explorativen Ergebnisse darauf hin, dass deren Einfluss stärker über individuelle Rückmeldungen als über regelmäßigen Videokonferenzen auf die eigenständige Bewältigung von Lernaufgaben wirken könnte – wobei insgesamt die direkte Kommunikation zwischen Lehrkräften und Schülerinnen und Schülern als nicht sehr ausgeprägt beschrieben wird. Die vermuteten positiven Einflüsse von längeren Lernzeiten und regelmäßiger elterlicher Unterstützung zeigen sich in den Daten dieser Stichprobe nicht. Vielmehr weisen die Ergebnisse dieser Stichprobe darauf hin, dass diejenigen, die mehr Zeit für die Aufgaben verwenden und regelmäßige Unterstützung durch Eltern benötigen, häufiger Schwierigkeiten bei der Bewältigung der Aufgaben haben – möglicherweise wird die familiäre Unterstützung und der Umfang der Lernzeiten dann gesteigert, wenn die Aufgabenbewältigung weniger gelingt.

## Diskussion

Der Beitrag sieht sich als ein Versuch einer Annäherung an Theorie und Empirie der Fernbeschulung und möchte zur wissenschaftlichen Durchdringung dieses Phänomens beitragen. Deutlich wird die historische Besonderheit der Situation, die (nicht nur) in Deutschland erstmals für einen gewissen Zeitraum alle Schülerinnen und Schüler betraf. Das entworfene Rahmenmodell für eine systematische Erfassung des Bedingungsgefüges von Fernunterricht orientiert sich dabei an einem klassischen Schulmodell und erweitert dies insbesondere um digitale Elemente, die in den verschiedenen Dimensionen des Modells zum Tragen kommen. Entsprechend erscheint auch das Thema E‑Learning, das bislang im Kontext der Schulforschung und Schultheorie noch wenig entwickelt ist, als wichtiger Baustein für ein Verstehen von Fernbeschulung. Dass deutsche Schulen hier im internationalen Vergleich sowohl in Ausstattung als auch in der digitalen Praxis Aufholbedarf haben, wurde bereits in der Sonderauswertung zu PISA 2018 (vgl. OECD 2020, S. 115 ff.) und in den Ergebnissen der ICILS-Studien (vgl. Eickelmann et al. 2019) formuliert. Zudem zeigt die Studie der Vodafone Stiftung Deutschland (2020), dass jene Schulen, die bereits vor den Schulschließungen digital besser aufgestellt waren, weniger Probleme in der Fernbeschulung hatten. Die Daten der vorliegenden Studie legen am Beispiel Lernplattformen die Hypothese nahe, dass es dabei nicht nur um die bloße Existenz, sondern auch um die Befähigung aller zum Umgang damit geht.

Die Bedeutung des Digitalen (Infrastruktur, Kommunikation, Lehrformen, IT-Kompetenz von Lehrenden und Lernenden), welche die hier präsentierten Studien zur Fernbeschulung im Jahr 2020 herausarbeiten, lässt sich auch mit den hier präsentierten Befunden stützen. Gerade die Computerfähigkeiten der Schülerinnen und Schüler, das digitale Feedback sowie die häusliche digitale Infrastruktur erweisen sich in der vorliegenden Studie als bedeutsam für die häusliche Aufgabenbewältigung. Dies findet seine Entsprechungen auch in den Studien aus dem Bereich E‑Learning, in dem vor allem IT-Kompetenzen der Lernenden als zentraler Erfolgsfaktor betrachtet wird (vgl. z. B. Selim 2007, S. 406).

Das hier vorgestellten Modell stützt zudem die Hypothese, dass die Fähigkeit zum selbstregulierten Lernen und die individuellen Computerfähigkeiten wichtige Faktoren für die Fernbeschulung sind, deren Entwicklung die Schulen mit Blick auf möglicherweise erneute Schulschließungen durch entsprechende Lehre noch stärker fördern könnten. Auch die Bedeutung der Kontakte zu Lehrpersonen und individueller Rückmeldungen in Zeiten der Fernbeschulung unterstreicht die Notwendigkeit, die Schülerinnen und Schüler (und ihre Eltern) nicht mit ihren Aufgaben allein zu lassen und vermehrt interaktivere Lehrangebote schaffen.

Auch die Befunde zur Gefahr größerer herkunftsbedingter Bildungsungleichheiten durch Fernbeschulung aus der eigenen und den referierten Studien bieten Anlass zu weiterer Forschung und politischer wie schulischer Aktion (vgl. auch Dancer et al. 2020), besonders dann, wenn Schulschließungen und Fernbeschulung Deutschland noch länger beschäftigen. Aufgrund des gymnasialen Bias der Stichprobe müssen Analysen dieser Untersuchung im Zusammenhang mit herkunftsbedingter Bildungsungleichheiten mit besonderer Vorsicht interpretiert werden. Die Daten der Stichprobe deuten dabei in die Richtung, dass zum einen eine nicht-deutsche Haushaltssprache die Bewältigung der Fernbeschulung erschwert. Des Weiteren zeigen sich in den Daten auch indirekte Herkunftseffekte, in dem die Faktoren niedrigerer Bildungsstand der Eltern und nicht-deutschen Haushaltssprache sich durch die damit einhergehende geringere digitale Geräteausstattung negativ auf die selbsteingeschätzte Möglichkeit zur Bewältigung der Aufgaben auswirkt. Diese Befunde unterstützen die artikulierte Dringlichkeit weitere empirisch gestützter, effektiver Maßnahmen zur besseren Ausgestaltung von Fernbeschulung mit Blick auf mögliche langfristige Folgen der Schulschließungen durch die Coronakrise (vgl. Anger et al. 2020b; Köller et al. 2020).

Dabei hat die hier vorgelegte Studie Grenzen, welche die Interpretation und Reichweite der Ergebnisse deutlich limitieren: zum einen durch einen zwar umfangreichen, aber doch auf acht Schulen begrenzten Datensatz, der keine Schlüsse auf eine Grundgesamtheit von Schülerinnen und Schülern erlaubt. Angenommen werden muss, dass über den mehrfachen Selektionsprozess eher motivierte Kinder und Jugendliche aus bildungsaffineren Haushalten erreicht worden sind, während jene, denen die digitale Infrastruktur fehlt oder über die Schulen nicht gut erreicht wurden, anteilig möglicherweise in der Studie unterrepräsentiert sind. Auch setzt die Studie allein auf die Selbstauskünfte von Kindern und Jugendlichen, die von vermeintlich objektiven Rahmenbedingungen innerhalb einer Schule durchaus abweichen (wie z. B. das Vorhandensein einer schulischen Lehrplattform). Zusätzlich zu den stichprobenbedingten Limitationen muss bei der Interpretation der Ergebnisse beachtet werden, dass das hier zugrunde gelegte sehr komplexe Modell nicht mit dem Anspruch einer vollständigen empirischen Überprüfung, sondern als Versuch einer umfangreichen theoretischen Systematisierung angelegt wurde. Dieses Modell wurde als explorativer Rahmen, der noch nicht in allen Zusammenhängen schon theoretisch und empirisch geprüft war, für die Analyse der eigenen Daten genutzt, die auch nicht alle Dimensionen abdecken und somit das theoretische Modell als Ganzes so nicht geprüft wird. Hierfür wäre sowohl eine noch breitere Datenerhebung und andere Stichproben erforderlich, die aber gerade in Zeiten von Schulschließungen nur schwer zu erreichen sind.

Aus dem vorgeschlagenen theoretischen Modell und der mit Blick auf die skizzierten deutlichen Limiationen der Stichprobe eher exploratorischen Funktion der vorliegenden Studie können jedoch Impulse für Instrumente und Inhalte von Folgestudien formuliert werden. So wären der Einbezug der Perspektive von *Eltern und Schulverantwortlichen/Lehrkräften* und die Erhebung von Indikatoren zur Qualifikation wie auch der Lehrformen bei diesen Personengruppen wichtige Schritte, um die Alltagsrealitäten der Fernbeschulung fundierter zu analysieren. Gerade in der Dimension der Lehrpersonen weisen die bisherigen Studien auf eine große Heterogenität der Ausgestaltung der Fernbeschulung zwischen den Lehrkräften hin (vgl. z. B. Vodafone Stiftung Deutschland 2020). Mit Blick auf das *Gelingen* von Fernbeschulung sind zudem die Wirkungen der Fernbeschulung noch systematischer zu betrachten, vor allem hinsichtlich des tatsächlichen *Lernerfolgs* für fachspezifische wie auch digitale Kompetenzen. Hier ließe sich bspw. das Vorgehen von Schwerdt und Chingos (2015) adaptieren, um zu prüfen, inwiefern sich Leistungen von Schülerinnen und Schülern in der digitalen Fernbeschulung von jenen unterscheiden, die denselben Stoff analog vor Ort in einer Schule lernten. Zu fragen wäre auch, inwieweit über Fernbeschulung die „anderen“ Funktionen der Schule, gerade mit Blick auf die persönliche Entwicklung und soziales Lernen, erfüllt werden können. Für solche und weitere Analysen wäre eine Stichprobe mit repräsentativem Zugang zu Schulen, Schulformen und Regionen wünschenswert, der auch die statistische Modellierung von Kontexten (auch in Fernbeschulung bleiben die Schülerinnen und Schüler in Klassenzusammenhängen der Schulen und erhalten klassenweise Aufgaben von ihren Lehrkräften) ermöglichen würde.

## Footnotes

1 Charter Schools sind nicht-staatliche Schulen, die im Zuge der Bildungsreformen unter dem „No Child Left Behind Act“ in 2001 entstanden sind (vgl. Sherbondy 2008, S. 7).

2 Dabei handelt es sich um öffentliche, gebührenfreie Institutionen, die nicht den gleichen geografischen Limitationen unterliegen wie herkömmlichen „Charter-Schools“ und daher Schülerinnen und Schüler erreichen, die auch weiter entfernt leben (vgl. Sherbondy 2008, S. 9).

3 Vgl. dazu auch Huber und Helm (2020, S. 248); Wößmann et al. (2020, S. 28 f.).

4 Eine diesem Punkt innewohnende Frage ist, wie sich Vorteile zeitunabhängigen Lernens und Vorteile von face-to-face Instruktionen zueinander verhalten.

5 Sander et al. (2020) ermitteln in ihrer Studie, dass bei insgesamt 60 % der Schülerinnen und Schüler nach Elternangaben eine „kompetenten Unterstützungsperson“ anwesend ist.

6 Auf Signifikanzangaben wurde aus oben benannten Gründen verzichtet.

7 Analog zu diesem Modell wurde ein äquivalentes Modell mit einer Skala zur Bewertung des Lernens in den Fächern Mathe, Deutsch und erster Fremdsprache geschätzt. Dieses erbrachte vergleichbare Pfadkoeffizienten und auch einen fast identischen Modelfit.
